# Epigenetics in Breast Cancer Therapy—New Strategies and Future Nanomedicine Perspectives

**DOI:** 10.3390/cancers12123622

**Published:** 2020-12-03

**Authors:** Verona Buocikova, Ivan Rios-Mondragon, Eleftherios Pilalis, Aristotelis Chatziioannou, Svetlana Miklikova, Michal Mego, Karlis Pajuste, Martins Rucins, Naouale El Yamani, Eleonora Marta Longhin, Arkadij Sobolev, Muriel Freixanet, Victor Puntes, Aiva Plotniece, Maria Dusinska, Mihaela Roxana Cimpan, Alena Gabelova, Bozena Smolkova

**Affiliations:** 1 Cancer Research Institute, Biomedical Research Center of the Slovak Academy of Sciences, Dubravska Cesta 9, 845 05 Bratislava, Slovakia; verona.buocikova@savba.sk (V.B.); svetlana.miklikova@savba.sk (S.M.); alena.gabelova@savba.sk (A.G.); 2 Department of Clinical Dentistry, University of Bergen, Aarstadveien 19, 5009 Bergen, Norway; ivan.rios-mondragon@uib.no (I.R.-M.); Mihaela.Cimpan@uib.no (M.R.C.); 3 e-NIOS Applications Private Company, Alexandrou Pantou 25, 17671 Kallithea, Greece; epilalis@e-nios.com (E.P.); achatzi@e-nios.com (A.C.); 4 Center of Systems Biology, Biomedical Research Foundation of the Academy of Athens, 11527 Athens, Greece; 5 2nd Department of Oncology, Faculty of Medicine, Comenius University and National Cancer Institute, Klenova 1, 833 10 Bratislava, Slovakia; michal.mego@nou.sk; 6 Latvian Institute of Organic Synthesis, Aizkraukles str. 21, LV-1006 Riga, Latvia; kpajuste@osi.lv (K.P.); rucins@osi.lv (M.R.); arkady@osi.lv (A.S.); aiva@osi.lv (A.P.); 7 Health Effects Laboratory, NILU-Norwegian Institute for Air Research, 2007 Kjeller, Norway; ney@nilu.no (N.E.Y.); eml@nilu.no (E.M.L.); mdu@nilu.no (M.D.); 8 Vall d Hebron, Institut de Recerca (VHIR), 08035 Barcelona, Spain; muriel.freixanet@vhir.org (M.F.); victor.puntes@vhir.org (V.P.); 9 Institut Català de Nanosciència i Nanotecnologia (ICN2), Bellaterra, 08193 Barcelona, Spain; 10 Institució Catalana de Recerca i Estudis Avançats (ICREA), 08010 Barcelona, Spain

**Keywords:** epigenetics, breast cancer, nanomedicine, epi-drugs, targeted delivery, drug resistance

## Abstract

**Simple Summary:**

Despite advances in cancer treatment, difficult-to-treat tumor subtypes remain a challenge. New multidisciplinary approaches can help overcome current obstacles posed by tumor heterogeneity, activation and enrichment of cancer stem cells, and acquired drug resistance development. Epigenome modulation, currently unsuccessful in solid tumors due to epigenetic drug instability, toxicity, and off-target effects, might be enabled by implementing nano-based delivery strategies aiming to improve breast cancer patient outcomes.

**Abstract:**

Epigenetic dysregulation has been recognized as a critical factor contributing to the development of resistance against standard chemotherapy and to breast cancer progression via epithelial-to-mesenchymal transition. Although the efficacy of the first-generation epigenetic drugs (epi-drugs) in solid tumor management has been disappointing, there is an increasing body of evidence showing that epigenome modulation, in synergy with other therapeutic approaches, could play an important role in cancer treatment, reversing acquired therapy resistance. However, the epigenetic therapy of solid malignancies is not straightforward. The emergence of nanotechnologies applied to medicine has brought new opportunities to advance the targeted delivery of epi-drugs while improving their stability and solubility, and minimizing off-target effects. Furthermore, the omics technologies, as powerful molecular epidemiology screening tools, enable new diagnostic and prognostic epigenetic biomarker identification, allowing for patient stratification and tailored management. In combination with new-generation epi-drugs, nanomedicine can help to overcome low therapeutic efficacy in treatment-resistant tumors. This review provides an overview of ongoing clinical trials focusing on combination therapies employing epi-drugs for breast cancer treatment and summarizes the latest nano-based targeted delivery approaches for epi-drugs. Moreover, it highlights the current limitations and obstacles associated with applying these experimental strategies in the clinics.

## 1. Introduction

The most common cancer diagnosed among women is breast cancer (BC), the second leading cause of cancer deaths [1]. Besides well-studied genetic changes, epigenetic alterations, resulting in aberrant gene expression, are among the key contributors to breast carcinogenesis. Different mechanisms introduce and maintain epigenetic modifications, including DNA methylation, post-translational histone modifications, and non-coding RNA-mediated regulation [2]. Epithelial-mesenchymal transition (EMT) is a complex developmental program, which plays a crucial role in the hematogenous and lymphatic dissemination of tumors. EMT facilitates phenotypic metamorphosis of epithelial tumor cells into highly motile and more aggressive mesenchymal cells that can colonize distant organs. Moreover, this multistep process enables the generation of tumors with stem cell properties, which play a significant role in developing therapeutic resistance [3]. The reversibility of EMT, allowing circulating tumor cells (CTCs) to remain epithelial in their origin, endowing them with a potential to seed metastasis, supports the hypothesis about its epigenetic regulation [4]. EMT is triggered by extracellular signals, including extracellular matrix proteins and soluble growth factors, or by intracellular cues. It is mediated by a group of pleiotropic transcription factors (TFs), which control a heterogeneous network of epigenetic effectors, thus allowing potent gene expression changes [4]. This epigenetic plasticity not only permits dynamic regulation of expression but also offers numerous therapeutic opportunities.

Currently, BC treatment involves a multidisciplinary approach. Although the effectiveness of various therapeutic regimens has increased, resulting in reduced mortality [5], there are still many obstacles to overcome. These include serious side effects, hard-to-treat tumor subtypes, intratumoral heterogeneity, and at present incurable metastatic disease. The success achieved so far in treating hematological malignancies using epigenetic inhibitors has stimulated interest in their use to treat solid tumors. Promising preclinical results suggest that epigenetic drugs (epi-drugs) can sensitize resistant cancer cells to traditional approaches. Unfortunately, these results have not yet been confirmed by clinical studies, as the early-generation epi-drugs were basically broad-spectrum reprogrammers, causing large-scale gene expression changes. This “one size fits all” approach has mostly failed due to off-target effects, significant toxicities, the risk of large-scale epigenomic repatterning, and the lack of appropriate biomarkers for patient selection. However, this failure has led to the development of selective new-generation epi-drugs, which, together with precision medicine design, provide a new chance for epigenetic therapy of solid tumors [6,7].

Among the options that could contribute to successful clinical applications of epi-drugs are new technologies for safer and more efficient cancer cell epigenome modulation. Advances in nanotechnology and material science have provided a broad variety of more precise and safer nanoscale organic and inorganic nanomaterials for drug delivery (e.g., dendrimers, micelles, liposomes, gels, metal- and carbon-based nanomaterials). To date, several nanomaterials have been successfully studied and introduced in cancer treatment, and many others are undergoing clinical trials. Encapsulation by intelligent nanocarriers of antitumor drugs, conventional chemotherapeutics, epi-drugs, or both, can improve their solubility and stability by protecting the drugs from fast clearance and degradation, thus prolonging their half-life in the systemic circulation [8]. Nanocarriers can also be tuned to ensure targeted, controlled, and sustained release, thereby reducing toxicity [8,9,10]. Nanoscale size and unique physicochemical properties (e.g., shape, surface area, and charge) allow the accumulation of nanocarriers in the tumor mass due to the enhanced permeability and retention (EPR) effect, which is the basis of passive targeting [11]. The spatial and temporal heterogeneity of tumors is one of the limitations of therapeutic efficacy in passive targeting [12]. Functional surface modifications of nanocarriers by specific ligands (antibodies, aptamers, proteins, etc.) with a high affinity for particular receptors overexpressed on the tumor cells allows active targeting of drug delivery to the tumor mass, thereby increasing treatment efficacy and reducing side effects [13]. The biggest challenge to combat BC is to eliminate cancer stem cells (CSCs) that play a crucial role in metastasis and the development of multidrug resistance to therapy. Nanoscale delivery systems represent a promising tool for their eradication [14].

In this review, we provide an outline of current achievements in epigenetic therapy of BC, focusing on the ability of epi-drugs to sensitize resistant cancer cells to standard therapeutic approaches. Furthermore, we highlight the promise of nanomedicine with regard to overcoming obstacles associated with the successful use of epi-drugs for the treatment of solid tumors.

## 2. Molecular Pathology of Breast Cancer

The BC incidence rate varies from 27.9 per 100,000 people in Middle Africa to 92.6 per 100,000 in Western Europe [1]. The differences in incidence are attributed to different risk factors and the availability of improved imaging techniques for screening and diagnosis [15]. The five-year relative survival rate for women diagnosed with the regional disease was recently estimated at 86%, whereas it was 27% for those with metastatic disease [16]. Therapeutic resistance and metastatic potential are influenced by the heterogeneity of phenotypic and molecular characteristics [17,18,19,20]. While the luminal A subtype is considered a low-grade disease with a good prognosis and likely to benefit from endocrine therapy alone, luminal B tumors have a higher proliferation rate, worse prognosis, and patients require additional chemotherapy treatment. The human epidermal receptor 2 (HER2)-overexpressing tumors tend to grow faster and can have higher histological grade than luminal-like tumors, but generally, they are successfully treated by targeted anti-HER2 therapies. Triple-negative/basal-like BC is a histologically high-grade disease associated with a poor prognosis. Patients with this subtype do not benefit from targeted therapies, and the standard chemotherapy regimen is the only suitable therapeutic approach at present.

### 2.1. Role of Epigenetics in BC Pathogenesis

Approximately 10% of BCs are considered to be hereditary. Most of them are associated with mutations in tumor suppressor genes *BRCA1* and *BRCA2* or other high or moderate-penetrance genes, such as *CHEK2, ATM, PALB2, PTEN, STK11*, and *TP53* [21]. In general, BC initiation, followed by histological progression from premalignant stages to invasive carcinoma, develops from the accumulation of genetic and epigenetic changes. These aberrations involve the inactivation of tumor suppressor genes and/or oncogene activation, enabling the continuous malignant transformation of cells [22]. The main genetic changes comprise point mutations (single nucleotide substitutions, small insertions, and deletions), structural rearrangements, and large scale copy number changes [23]. The most frequently altered genes reported in early BCs are *TP53*, *PIK3CA*, *MYC*, *PTEN, CCDN1*, *ERBB2*, *FGFR*, and *GATA3* [24].

Apart from genomic alterations, cancer initiation and progression are driven by the combined action of multiple epigenetic changes [25]. The term epigenetics describes heritable DNA modifications that do not change the DNA sequence but can affect gene expression. Epigenetic regulation is involved in many normal cellular processes, including cell growth and differentiation [26]. The major epigenetic modifications encompass changes in DNA methylation, post-translational histone modifications, and non-coding RNA expression. These modifications are thought to participate in early BC carcinogenesis events and can be useful as biomarkers for early detection and the determination of prognosis and response to treatment [27].

#### 2.1.1. DNA Methylation

DNA methylation is the chemical modification caused by covalent attachment of a methyl group at cytosine within CpG dinucleotides, resulting in the formation of 5-methyl-cytosine (5mC). The methyl group transfer from the S-adenosyl-L-methionine donor is catalyzed by three DNA methyltransferases (DNMTs), DNMT1, DNMT3A, and DNMT3B. DNMT1 maintains existing methylation patterns during replication, while DNMT3A and DNMT3B are responsible for de novo methylation [28]. DNA methylation occurring in the promoter regions inhibits gene expression by preventing the binding of the transcriptional machinery to their recognition sequences or by binding proteins with a methyl-binding domain (MBD) that have a higher affinity to promoters [29]. MBD proteins interlink DNA methylation and histone modifications by engaging histone deacetylase complexes and chromatin remodeling factors, leading to chromatin condensation and thus to transcriptional repression [30].

Aberrant DNA methylation is a hallmark of cancer. The methylation of normally unmethylated promoter CpGs, known as hypermethylation, can lead to tumor suppressor genes’ inactivation, thus acting as a potential biomarker for early cancer detection and prognosis [31]. Furthermore, DNA hypomethylation on a genome-wide scale, reported in many cancers, can induce genomic instability [32]. De Almeida et al. identified 368 differentially methylated individual CpG sites in BC tumors compared to healthy breast tissues [33]. Hypermethylated CpG sites were mostly present in upstream promoter regions (56%), while hypomethylated CpG sites were localized mostly in the gene bodies (66%). Nevertheless, in many cancers, CpG island shores, the regions of low CpG density flanking traditional CpG islands (up to 2 kb distant) show distinct subtype-specific methylation signatures [34]. Lately, more than 100 genes have been found to be hypermethylated in BC [35]. They play essential roles in various cell mechanisms, including DNA repair (e.g., *MGMT*, *BRCA1*, *MLH1*), cell-cycle regulation (e.g., *CCND2*, *AK5*, *FOXA2*), apoptosis (e.g., *BCL2*, *APC*) [36], cell adhesion (e.g., *CDH1*), tissue invasion and metastasis (e.g., *RASSF1A*, *RARβ*, *TWIST*, *HIN1*), and hormone-mediated cell signaling (*ESR1*, *ESR2*, and *THRB*) [37].

For a long time, DNA methylation was referred to as an irreversible epigenetic event, which could only be passively depleted through DNA replication. However, this consideration changed with the discovery of ten-eleven translocation (TET) proteins [38]. The TET family, comprising TET1, TET2, and TET3 proteins, oxidize 5mC to 5-hydroxymethylcytosine, 5-formylcytosine, and 5-carboxylcytosine and accordingly facilitates active DNA demethylation [32]. TET proteins bind preferentially to unmethylated CpGs within CpG islands, thus maintaining CpG islands in a hypomethylated state, associated with active transcription [39]. Besides, decreased 5-hydroxymethylcytosine and loss-of-function mutations of TET proteins can potentially affect malignant transformation [40].

#### 2.1.2. Histone Modifications

Chromatin is a dynamic structure composed of nucleosomes. The main components of the nucleosomal subunit are the histones. These alkaline proteins form an octamer consisting of two identical subunits, each containing four histones: H2A, H2B, H3, and H4. The nucleosome core particle includes 147 bp of DNA sequence, wrapped in nearly two superhelical turns around a histone octamer [41]. In general, histones are highly conserved proteins that can be post-translationally modified at the amino acid residues located on their C- and N-terminal tails [42]. Post-translational histone modifications (PTMs) do not affect the DNA sequence but can influence gene expression by changing chromatin structure from the non-condensed transcriptionally active state (euchromatin) to the condensed inactive state (heterochromatin). Histone tail residues can undergo covalent PTMs, including methylation, acetylation, phosphorylation, sumoylation, glycosylation, ubiquitination, ADP-ribosylation, and carbonylation [43,44]. For instance, acetylated histone H3 and di- or tri-methylated histone H3 lysine 4 (H3K4me2, H3K4me3) in the promoter region result in the activation of gene expression. Conversely, repression of the promoters is usually caused by histone deacetylation and by tri-methylated histone H3 lysine 27 (H3K27me3) and tri-methylated histone H3 lysine 9 (H3K9me3) [45,46]. It is not surprising that many aberrations in histone modifications are found in cancer due to the essential roles of these modifications in DNA-mediated cellular processes. Furthermore, it has been shown that global loss of acetylation on lysine K16 and tri-methylation on lysine K20 of histone H4 is a hallmark of various cancers [47]. In BC, reduced levels of lysine acetylation (H3K9ac, H3K18ac, and H4K12ac), lysine methylation (H3K4me2, H4K20me3), and arginine methylation (H4R3me2) have been observed in poor prognostic tumors. Additionally, down-regulation of H4K16ac and the corresponding enzyme histone acetyltransferase (HAT) hMOF, in most primary breast tumors, represent an early sign of BC [48,49].

Different enzymes catalyze histone PTMs, including histone deacetylases (HDACs), which remove acetyl groups, histone methyltransferases (HMTs), demethylases (HDMs), HATs and others. At least 18 HDACs have been identified in humans and classified into four groups [50]. Histone acetylation marks are read by bromodomain protein modules, which are evolutionarily conserved domains of 110 amino acids within many chromatin-associated proteins (including HATs) and other effector enzymes [51]. HATs are categorized into five families: GNAT, MYST, p300/CBP, SRC, and TAFII250. Members of the bromodomain family, bromodomain and extra-terminal domain (BET) proteins can increase proliferation and may potentiate the overexpression of several oncogenes such as *MYC* [52]. HMTs, along with HDMs, introduce or remove methyl groups and modify other proteins in addition to histones [53].

Molecular changes that affect the expression of histone-modifying enzymes could also contribute to cancer development and progression. In BC, various alterations have been identified, including overexpression of p300, HBO1, HDAC1, HDAC2, HDAC3, HDAC6 [54], amplification and overexpression of enhancer of zeste homolog 2 (EZH2) [55], depletion of H3K9 trimethyl-demethylase (JMJD2B) [56], downregulation of lysine-specific histone demethylase 1A (LSD1) [57], and others. Due to more demanding methodological approaches, the role of histone modification in cancer is less studied than DNA methylation or changes in non-coding RNA (ncRNAs).

#### 2.1.3. Epigenetic Regulation by Non-Coding RNA

The ncRNAs are molecules of RNA that are not translated into proteins. However, they play an important role in epigenetic modifications. RNAs with regulatory functions are divided into two major classes based on their size: short-chain ncRNAs (including siRNAs, miRNAs, and piRNAs) less than 200 nucleotides in length, and long ncRNAs (lncRNAs) [58]. The ncRNAs can directly influence gene expression or be epigenetically regulated themselves [59]. Alterations in their expression contribute to the pathogenesis of most human cancers [60,61,62].

MicroRNAs (miRNAs) are endogenous, short, single-stranded RNAs, 19–25 nucleotides in length, which regulate gene expression by post-transcriptional silencing or the degradation of target mRNAs [61]. In general, they can be categorized into tumor-promoting (oncomiRs) and tumor-suppressing miRNAs. During carcinogenesis, oncomiRs are usually amplified, and tumor-suppressing miRNAs are down-regulated [63]. miRNAs can be readily identified in tumor samples (non-circulating miRNAs) and different body fluids (circulating miRNAs). Deregulation of miRNAs is involved in each cancer hallmark, such as proliferation, invasion and metastasis, apoptosis, etc. [64,65].

In BC, the expression profiles of miRNAs have been determined as promising biomarkers for diagnosis, more precise subtyping, prognosis, and therapy response [66]. It was observed that luminal A tumors have higher expression of the miR-99a/let-7c/miR-125b-2 miRNA cluster compared to luminal B tissues [67]. The miR-1, miR-92a, miR-133a, and miR-133b were determined as essential diagnostic markers by analyzing miRNA profiles between tumor and serum samples [68]. Furthermore, it was shown that patients with up-regulated expression of miR-1307-3p, miR-940, and miR-340-3p had a worse overall survival [69], while miR-21 and miR-205 were associated with disease-free survival interval and miR-205 with overall survival [70]. In addition, miRNA-449a overexpression was found to have propitious prognostic significance in BC patients [71].

### 2.2. Epigenetics in BC Progression

EMT is a fundamental process having a crucial role during embryogenesis and tissue regeneration. EMT, together with the mesenchymal-epithelial transition (MET), is required to form organs and cellular structures [72]. Moreover, they are involved in malignant progression, where EMT is responsible for the loss of cellular adhesion, cytoskeleton remodeling, acquisition of cancer cells’ migratory capacity as well as their ability to intravasate and survive in circulation [73]. Typical phenotypic EMT stages range from fully differentiated epithelial to dedifferentiated mesenchymal cells. EMT is triggered by extracellular agents such as growth factors (e.g., TGF-β [74]) and by non-growth factor stimuli (e.g., hypoxia, oxalate, Galectin-8 [72]). Stromal constituents of the tumor microenvironment can activate EMT via secretion of various cytokines and chemokines acting in a paracrine fashion on nearby carcinoma cells. Tumor-associated stromal cells include cancer-associated fibroblasts, regulatory T cells, CD4+ helper T and CD8+ cytotoxic T cells, tumor-associated macrophages, and myeloid-derived suppressor cells. These cells can promote tumor progression and metastasis. Moreover, activation of EMT can contribute to the immunosuppressive tumor microenvironment, which has been observed in BC [75,76]. In mammary epithelial cells, the up-regulation of TWIST1 or SNAI1-induced formation of neoplastic cells with CSC properties, which are associated with a more aggressive basal-like BC subtype.

During EMT, epithelial cells down-regulate the expression of several cell adhesion molecules, among them E-cadherin and β-catenin. EMT-inducing TFs play a critical role in this process. While several of them are well known, notably SNAI1/2, TWIST1/2, ZEB1/2, FOXC1/2, and TCF3, others may yet be discovered [77]. Until recently, the down-regulation of E-cadherin has been considered a hallmark of EMT; however, this assumption seems to be too simple in light of recent discoveries. For instance, Padmanaban et al. showed that E-cadherin is required for invasive ductal carcinoma cell survival and metastasis [78]. Although E-cadherin loss was associated with increased invasion into local tissue, in parallel, it was connected with a reduction in cell growth and survival, as well as a decreased amount of CTCs in the peripheral blood of patients. Notably, cell clusters rather than individual invading cells are involved in the initiating steps of malignant progression. E-cadherin is expressed in the cells that form these clusters [79]. Thus, the notion that E-cadherin is repressed during EMT contradicts recent findings, which have helped to explain why E-cadherin expression is often observed in the BC tumors and metastases. Although several reports describe the occurrence of metastases formed by cells that never underwent EMT [80], these studies have been criticized for insufficient experimental evidence [81].

While EMT is driving the dissemination of cancer cells, MET is crucial for metastatic colonization [82]. MET can occur as a passive consequence of the down-regulation of EMT-TFs or their active repression [83]. In BC, MET can be promoted by TFs such as Prrx1 or ZEB1/2 or the expression of ID1 and ID3 genes [84]. In the past few years, evidence has accumulated to demonstrate that the phenotypic plasticity of the metastatic process is mediated via epigenetic modifications driven by these TFs. After their binding to promoters and enhancers, they interact with epigenetic regulators and chromatin remodeling machinery to up-regulate the expression of pro-mesenchymal genes and down-regulate epithelial genes [85]. These DNA-binding proteins act as intermediaries between cellular signaling and chromatin remodeling, thus guiding the epigenetic machinery to their target sites.

It has been demonstrated that during EMT, HMT G9a, which can also methylate non-histone proteins, interacts with Snail to recruit it to the CDH1 promoter [86,87]. A number of these TFs contain methyl-lysine binding motifs, crucial for correct chromatin recognition, supporting their role in EMT´s epigenetic regulation. It was shown that Zeb2 inhibits E-cadherin via DNA methylation [88]. Hypoxia-induced EMT is regulated via HDACs, namely HDAC3, coupled with H3K4Ac, present at promoter regions of traditional EMT genes such as CDH1 and VIM. Chromatin changes are also responsible for TGF-β mediated EMT, where the new chromatin modifiers were identified, e.g., UTX, PRMT5, Rad21, or RbBP5 [89]. Cells that have undergone EMT can also acquire drug resistance and stem cell-like properties [90]. Traditional EMT-associated TFs were shown to confer resistance to oxaliplatin-based and cisplatin-based chemotherapeutics, while the loss of E-cadherin was associated with resistance to several growth factors and kinase inhibitors [91,92].

Given their mode of action, epigenetic inhibitors can restore aberrant DNA methylation or histone deacetylation or impede the recognition of acetylated lysine residues and, therefore, potentially re-establish epithelial phenotypes during EMT. Comprehensive bioinformatic analysis has identified and validated various drug combinations together with HDAC inhibition that hamper EMT [93,94]. Thus, EMT, the crucial step in cancer progression and metastasis, can be potentially modulated by epi-drugs.

## 3. Breast Cancer Therapeutic Opportunities

### 3.1. The Biomarker-Directed Approach in BC Treatment

To overcome the limitations incurred by resistance mechanisms in the clinical management of advanced cancers, an increasing effort is being made towards biomarker-driven cancer treatments. This aims to identify important biomarkers capable of addressing tumor heterogeneity and effectively predicting a favorable clinical outcome as a response to a particular treatment. BC, in particular, is considered a family of distinct diseases with a varying molecular basis. Initial gene expression studies using cDNA microarrays have resulted in a classification of BC into five subtypes, establishing as major BC biomarkers estrogen receptor (ER), progesterone receptor (PR), and HER2 [95,96]. Therefore, currently, there is a mandatory need to define their expression status, lymph node involvement, and tumor size for all patients with invasive BC for therapy decision making. These markers are established in international guidelines as essential factors for the clinical management of primary BC patients [15]. However, the stratification of patients based solely on ER, PR, and HER2 expression has proven inefficient, unable to capture the substantial phenotypic complexity and heterogeneity of BC, thus stressing the need to integrate additional biomarkers for a more refined characterization. Ki-67, a non-histone nuclear protein, is used as a marker of cell proliferation. Ki-67 expression is significantly higher in malignant tissues with poorly differentiated tumor cells than in normal tissue and is thus used to assess tumor aggressiveness [97].

Traditional therapy of non-metastatic BC involves multidisciplinary strategies combining surgery, radiotherapy, neo-/adjuvant, endocrine, and targeted therapy [98]. For non-metastatic BC, the primary therapy approach consists of eradicating tumor and regional lymph nodes and preventing metastatic relapse. The first two goals are usually achieved by locoregional therapy that involves surgery and radiotherapy and/or neoadjuvant therapy in case of locally advanced disease. Prevention of metastatic relapse is achieved with systemic therapies that comprise anthracycline and taxane-based chemotherapy, anti-estrogen hormonal therapy, and anti-HER treatment, depending on receptor status [99].

In contrast to early BC, metastatic disease (stage IV), with common sites of spread in bones, brain, lung, and liver, is considered incurable, and the therapy aims to prolong life while minimizing symptoms or side effects. The combinations of endocrine, targeted therapy, chemotherapy, and immunotherapy can be administered to the metastatic patients, taking into account the tumor subtype, extent, and localization of the disease and the presence of specific molecular alterations. Beyond HER2 and ER/PR, new predictive biomarkers for targeted therapy in metastatic BC include *BRCA1/2* and *PI3KCA* mutations for PARP and PI3KCA inhibitors, respectively, and PD-L1 expression and/or MSI status for immunotherapy. The effective new biological therapies like CDK4/6 or mTOR inhibitors are now emerging. However, we still lack predictive biomarkers for these treatments [100].

### 3.2. Precision Medicine Concept

The maturation of omic technologies as powerful molecular epidemiological screening tools has empowered the emergence of manifold predictive biomarker signatures. The integration of genomic and transcriptomic profiles of 2000 breast tumors from the METABRIC (Molecular Taxonomy of Breast Cancer International Consortium) cohort revealed ten BC subtypes, termed integrative clusters (IntClust/s) and characterized by distinct genomic drivers [101]. Currently, there are five main standardized genetic prognostic platforms for BC, aiming to assist decision on therapeutic options, mainly including hormone therapy, chemotherapy, and anti-HER2 treatment:

- Oncotype DX provides prognostic information in terms of 10-year distant recurrence. It predicts the likelihood of adjuvant chemotherapy benefit in ER+ BC patients, based on the expression of a panel of 21 genes (16 cancer-related and five reference genes) [102].
- Breast Cancer Index assesses the expression of 7 genes to predict the benefit from extended, adjuvant, endocrine therapy (Tamoxifen) in HR+ patients. It is a gene expression signature comprising two functional biomarker panels, the molecular grade index (MGI) and the two-gene ratio *HOXB13/IL17BR* (H/I), that evaluate tumor proliferation and estrogen signaling, respectively [103]. MGI is a gene expression assay, measuring the expression of five genes (*BUB1B, CENPA, NEK2, RACGAP1, RRM2*) related to histological grade and tumor progression, which recapitulates tumor grade and can predict the clinical outcome with high performance [104].
- EndoPredict (Myriad Genetics, Inc., Salt Lake City, UT, USA) is a genomic test for people newly diagnosed with early-stage, ER+, HER2-negative BC (node-negative). It assesses the expression of 12 genes (8 target genes, 3 normalization genes, and 1 control gene) to predict response to chemotherapy [105].
- MammaPrint (Agendia, Irvine, CA, USA) is a 70-gene signature test that predicts the clinical outcome/response to chemotherapy in ER+ early-stage BC [106].
- Prosigna Breast Cancer Prognostic Gene Signature Assay (Nanostring, Seattle, WA, USA), formerly PAM50, assesses Tamoxifen response for HR+ BC patients based on the expression of 58 genes after 5 years of hormonal therapy treatment in postmenopausal women [107].

Besides Prosigna, Nanostring has developed a more extensive assay, the human nCounter Breast Cancer 360 panel, which comprises 776 genes across 23 key BC pathways and processes. Results are grouped in 48 signatures across 13 categories, measuring biological variables crucial to BC tumor biology. This panel has been developed for the evaluation of diverse BC aspects, including BC subtyping (luminal A/B, HER2-enriched, basal-like, triple-negative), expression of BC receptors and signaling (ESR1, PGR, ERBB2, AR, PTEN, CDK4, CDK6), mutational content (HRD, BRCA, P53), markers for tumor proliferation, apoptosis and differentiation (FOXA1, SOX2), cell adhesion (claudin), and immunity (chemokines, TGF-β, PD-1). Although a detailed description of the nCounterBC 360 panel exceeds this review’s scope, it contains a vast number of gene expression markers and indicates the intense molecular heterogeneity characterizing the diversity of BC phenotypes. Therefore, investigating the epigenetic landscape of BC may provide an additional layer of information that could improve our fundamental understanding of BC´s molecular complexity and the putative rational development of more effective and precise treatments.

Finally, various studies have identified and suggested epigenetic modifications and regulators as prognostic biomarkers for BC [108,109,110]. In this context, miRNAs have been considered a pool of highly potent biomarkers, as they have been linked to the identification of distinct molecular subtypes and tumor-related processes. miRNA expression profiling was successfully employed to classify the breast tumors as luminal A, luminal B, basal-like, HER2+, and normal-like BC [111]. Aberrant expression of miRNAs has also been correlated with clinical features, such as angiogenesis, metastasis, and EMT [112]. Besides, several lncRNAs have been reported as promising biomarkers for prognosis, diagnosis, and therapy [113]. Overall, there is cumulative evidence focusing on the systematic screening of epigenetic signals as a promising area for the discovery of novel molecular BC biomarkers, combining sensitivity, specificity, and robustness, with a potentially decisive impact on improving the quality of BC treatments in the context of precision medicine.

### 3.3. Potential of Epigenetic Therapy

**Figure 1:**
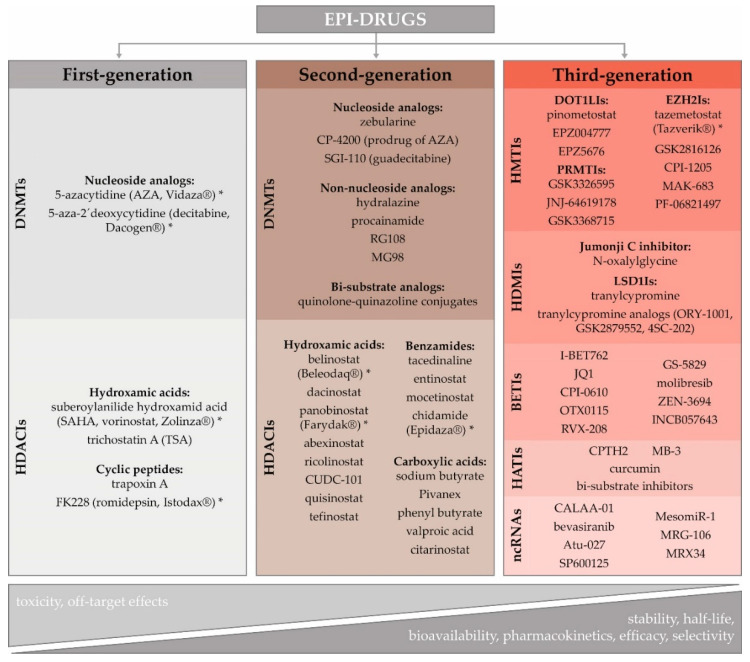
Different categories of epi-drugs, assessed in preclinical studies and clinical trials. Eight of them (indicated by asterisks) were approved to treat several human malignancies (modified from 7,119,120). Abbreviations: DNMTIs-DNA methyltransferase inhibitors; HDACIs-histone deacetylase inhibitors; HMTIs-histone methyltransferase inhibitors; HDMIs-histone demethylase inhibitors; BETIs-bromodomain and extra-terminal domain inhibitors; HATIs-histone acetyltransferase inhibitors; ncRNAs-non-coding RNAs; DOT1LIs-DOT1-like histone lysine methyltransferase inhibitors; EZH2Is-enhancer of zeste homolog 2 inhibitors; PRMTIs-protein arginine methyltransferase inhibitor; LSD1Is-lysine-specific histone demethylase 1A inhibitors.

The role of epigenetics in cancer initiation and progression, including its contribution to the development of innate and acquired resistance to several therapeutic regimens, has led to the scientific effort to reverse the aberrant cancer epigenome [32]. The lack of knowledge about subtype-specific epigenome signaling pathways, and missing patient-specific epigenetic biomarker profiles, are currently the main challenges hampering the wider clinical application of epigenetic agents in the treatment of solid cancers [114]. Over the last decade, several epi-drugs have received US Food and Drug Administration (FDA) approval for the treatment of blood-borne cancers: 5-azacytidine (AZA, Vidaza^®^), 5-aza-2´-deoxycytidine (decitabine, DAC, Dacogen^®^), vorinostat (VOR, SAHA, Zolinza^®^), romidepsin (FK228, Istodax^®^), belinostat (Beleodaq^®^), panobinostat (Farydak^®^), and chidamide (Epidaza^®^) (Figure 1) [115,116,117]. However, except for tazemetostat (Tazverik^®^), approved by FDA in January 2020 for metastatic or locally advanced epithelioid sarcoma, there is no epigenetic therapy approved for solid tumors, which are considered more epigenetically complex. Moreover, they exhibit abnormal vascularization, a specific tumor microenvironment, and more differentiated cells with decreased epigenetic reprogramming [7,118].

Rational epi-drug discovery using validated targets is a recent phenomenon. During early efforts, epi-drug development has been based on the demonstration of efficacy and phenotypic observations rather than on knowledge of their molecular targets. The timeline and key events influencing epi-drug development, including challenges and opportunities associated with their implementation in clinics, have recently been reviewed in-depth by Ganesan and colleagues [119]. As a detailed characterization of epi-drugs is beyond this paper’s scope, we provide only their basic characteristics and classification (Figure 1).

Given that overexpression of DNMTs and HDACs are considered the critical factors in carcinogenesis, demethylating agents and HDAC inhibitors (HDACIs) seem to be promising anticancer drugs [120,121,122]. Cytidine analogs, AZA and DAC, were the first DNMT inhibitors (DNMTIs) approved by the FDA in 2004 for the treatment of myelodysplastic syndrome and acute myeloid leukemia [123]. Although they were initially defined as cytotoxic agents, their therapeutic properties were achieved at lower doses and with prolonged exposure [124]. In general, HDACIs block histone deacetylation, causing reactivation of tumor suppressor genes that can inhibit cancer cell proliferation. Moreover, they have been shown to induce cancer cell death at concentrations to which normal cells are relatively resistant [125,126]. Chemically, HDACIs are classified into different subgroups: carboxylic acids, benzamides, cyclic peptides, and hydroxamic acids [127]. Similar to DNMTs, the first-generation HDACIs were characterized by poor bioavailability, low stability, and short half-life.

The development of second-generation epi-drugs has, therefore, been aimed to circumvent these shortcomings. Guadecitabine, a second-generation DNMTI, has a novel molecular structure, which prolongs its in vivo half-life, and increases efficacy [128]. As nucleoside analogs require active DNA synthesis to incorporate them into the DNA, their use is limited in hypoproliferative cancers and could be a major obstacle in the therapy of solid tumors [129]. Their common side effects could be avoided using non-nucleoside analogs, such as hydralazine, procainamide, RG108, and MG98 [130]. Recently, more efficient bi-substrate analogs have become potent DNMTIs [119]. Although the second-generation HDACIs, such as hydroxamic acid, belinostat, panobinostat, chidamide, or valproic acid, possess improved pharmacological properties, they achieved limited efficacy as single agents. However, their combination with other therapeutic approaches has allowed new avenues of their clinical investigation [7].

The principle of precision medicine is now being applied to the development and the use of third-generation epi-drugs, defined by a high degree of selectivity. This family includes HMT inhibitors (e.g., EZH2, DOT1-like histone-lysine methyltransferase (DOT1L), G9a and PRMT inhibitors), HDM inhibitors (e.g., LSD1 or Jumonji C domain inhibitors), BET inhibitors (BETIs) and HAT inhibitors (HATIs) [7,131]. HMTIs are emerging therapies targeting specific modifications. For example, it has been found that mutations in lymphomas activate the H3K27 histone methyltransferase EZH2, leading to disease progression. Therefore, the EZH2I can selectively target and induce cell death in cell lines with these mutations [132]. DOT1L is the only histone methyltransferase that targets the histone H3 lysine 79 (H3K79) residue [133]. Aberrant H3K79 methylation is associated with aggressive mixed-lineage leukemia and poor patient prognosis in lung, colorectal, and BCs [134]. This suggests that pharmacological inhibition of DOT1L can have therapeutic potential in several cancer types. The H3K4 and H3K9 demethylase enzyme LSD1 has an essential regulatory role in cell proliferation [135]. Its overexpression in several tumors has been correlated with a worse prognosis [136]. LSD1 inhibition may slow down cell growth in LSD1-overexpressing tumor cells. BETIs, JQ1, and I-BET762, are cell-permeable agents that reversibly and specifically bind the bromodomain proteins, thus impeding their interaction with acetylated histone lysine residues. It has been shown that they inhibit proliferation and induce apoptosis in various cancer cells [137]. HATIs include peptides, small molecules derived from natural products (e.g., curcumin), and synthetic molecules [138]. Peptide-CoA bisubstrate inhibitors mimic the formation of the substrate and cofactor complex binding to the HAT enzyme [131]. Well-conceived computational strategies and new screening platforms will be needed to predict loci specific epi-drugs sensitivities.

The ncRNAs, like miRNAs or siRNAs, with their power to selectively “switch-off” specific cancer genes, are attractive targets for the development of personalized cancer therapy. The main hindrance to the implementation of ncRNA-based therapy in clinical practice is the absence of effective delivery systems that can protect the RNA molecules from fast nuclease degradation before delivering them into the target cells´ cytoplasm [139]. There are two different possibilities to use miRNAs as therapeutic agents; substitution of depleted miRNAs (MRX34, miR34a replacement) and inhibition of overexpressed miRNAs by antagonistic oligonucleotides [140]. The inhibitor of miR-155, MRG-106, has been successfully investigated in phase I clinical trials for the treatment of hematological malignancies [141]. The miRNA-based therapeutic strategy also has great potential to regulate lncRNAs. The siRNA-mediated silencing by oligonucleotide inhibitors results in the inhibition of lncRNA-protein interactions and secondary structure changes, thus competing for their binding partners [142]. Down-regulation of cancer-related genes by siRNAs, e.g., CALAA-01 (targeting *RRM2*), Atu-027 (targeting PKN3), has been assessed in phase I/II clinical trials [139].

Although several ongoing clinical trials, including epi-drugs, exist on a wide range of diseases, many obstacles remain to be resolved. Among them are enzyme isoform selectivity, dual substrates, multimeric enzyme complexes involved in epigenetic regulations, high-order chromatin structure, functional effects of inhibition, and off-target effects. Other challenges are the pharmacology of the compounds, doses to be used, therapeutic regimens or duration of the treatment, and patient selection.

#### Epigenetic Therapy in BC

Several clinical studies have investigated the efficacy of DNMTIs (DAC, AZA) and HDACIs (VOR, phenyl butyrate) administration as BC monotherapy. However, these epi-drugs have shown limited antitumor efficacy at the maximum tolerated dose, suggesting the unsuitability of this approach [143]. In BC, the epi-drugs have been investigated in combination with cytotoxic agents, radiotherapy, targeted and hormone therapy, immunotherapy, as well as the combination of epi-drugs themselves (Table 1).

The DAC-chemotherapy combination has been widely studied in BC preclinical studies, and the results suggest enhanced sensitivity compared to cytotoxic therapy alone [144,145,146]. Despite these findings, clinical trials have been disappointing due to systemic toxicities and limited efficacy. Among the reasons leading to unsatisfactory results were the design of clinical trials using unselected patient populations and high-dose administration. This has revealed a need to identify appropriate epigenetic biomarkers for allowing a personalized approach and targeted delivery of epi-drugs [7].

The preclinical data with HDACIs on HR+ BC cell lines indicate an increase in antiproliferative endocrine therapy action [147]. In ER- BCs, an AZA-entinostat combination, and HDACI therapy (entinostat, valproic acid, TSA) alone, have shown a high ER re-expression and efficient restoration of the sensitivity to antioestrogen treatment [148,149,150,151]. Furthermore, the BET inhibitor JQ1 alone or in combination with the selective ER down-regulation by fulvestrant can effectively suppress the growth of tamoxifen-resistant cells [152]. The synergy of combination therapy has been preclinically demonstrated on triple-negative BC cells with mTOR-BETIs [153] and with PARP-HDACIs [154]. Based on promising preclinical results, the joint efficacy of HDACIs and anti-HER2 therapy with trastuzumab has been assessed in a clinical study (NCT00258349). Unfortunately, the hypothesis of reversing trastuzumab resistance by adding VOR in the therapeutic regimen has not been confirmed [155]. However, with the appropriate drug combination, it could be possible to target several oncogenic pathways [156].

All in all, for the successful utilization of epi-drugs in clinics, there is an urgent need for identification of new epigenetic therapeutic targets and mechanisms ensuring prolonged stability of epi-drugs, decreasing undesirable side effects while allowing increased efficacy via targeted delivery.

## 4. Nanomedicine as a Tool to Overcome the Current Limitations of Epigenetic Therapy

Nanomedicine applies the knowledge from material science and nanobiotechnologies to healthcare in order to improve the diagnosis, imaging, monitoring, prevention and regeneration, and to increase the efficacy and safety of the treatment [157]. According to the European Commission definition, a nanomaterial is a natural, incidental, or manufactured material with one or more external dimensions in the nanoscale (size range 1 nm–100 nm), and having a volume-specific surface area larger than 60 m^2^/cm^3^ [158]. The term nanomaterial in nanomedicine is broader and includes particles with dimensions up to 1000 nm [159]. Besides, the International Organization for Standardization has defined a nanoparticle (NP) as a nanomaterial, with the size in all three dimensions in the nanoscale [160]. However, these varied definitions and classifications allow a wide interpretation. Notably, the beneficial or detrimental potential of nanomaterials for the environment and human health depends on their physical and chemical properties, such as shape, composition, size, surface charge, and electron transfer [161].

### 4.1. Smart Nanoformulations for Drug Delivery Applications

Nowadays, a central area for the use of nanomedical products, considering the number of scientific publications and product approvals by regulatory agencies, is cancer treatment [162]. The conventional cytotoxic anticancer therapy is often associated with low therapeutic efficacy and increased systemic toxicity. The chemotherapeutic agents also affect rapidly dividing non-cancerous cells, causing common (nausea, vomiting, fatigue, hair loss) and drug-specific (e.g., cardiotoxicity, neurotoxicity, nephrotoxicity) side effects. Besides that, tumor cells have several defense mechanisms against the cytotoxic effect of chemotherapy. The most significant one is the multidrug resistance resulting from the overexpression of ATP-dependent efflux pumps with broad drug specificity [163].

Innovative materials, including NPs, with their unique properties, have several advantages as potential transport vehicles for standard and experimental anticancer agents. They allow encapsulation of hydrophobic and lipophilic molecules, enhancing their pharmacokinetics and circulation time in the body, leading to increased drug accumulation in the tumor site via passive targeting by the EPR effect. Moreover, they can be conjugated with specific ligands to bypass biological barriers and deliver a high drug concentration into the target tissue via active targeting. They also allow the drug´s release in a stable and controlled manner, triggered by pH, temperature, redox potential, etc. [164,165]. However, the efficient penetration of anticancer agents encapsulated in NPs remains an important issue in solid tumor therapy [166]. Systemically administered NPs should range from 10 to 200 nm in size to avoid early elimination by kidneys or subsequent entrapment by spleen and liver [167]. Biocompatibility, low toxicity, and low immunotoxicity are the prerequisite attributes of all biomedical nanomaterials. Before being approved for clinical use by the FDA, they have to undergo complex preclinical and clinical testing to confirm their biosafety. FDA-approved materials are, therefore, preferentially employed for the development of innovative nanocarriers. There is an increasing body of evidence from preclinical studies that the encapsulation of existing epi-drugs into different formulations has resulted in their improved stability and enhanced targeted delivery while minimizing off-target effects [168,169].

**Figure 2:**
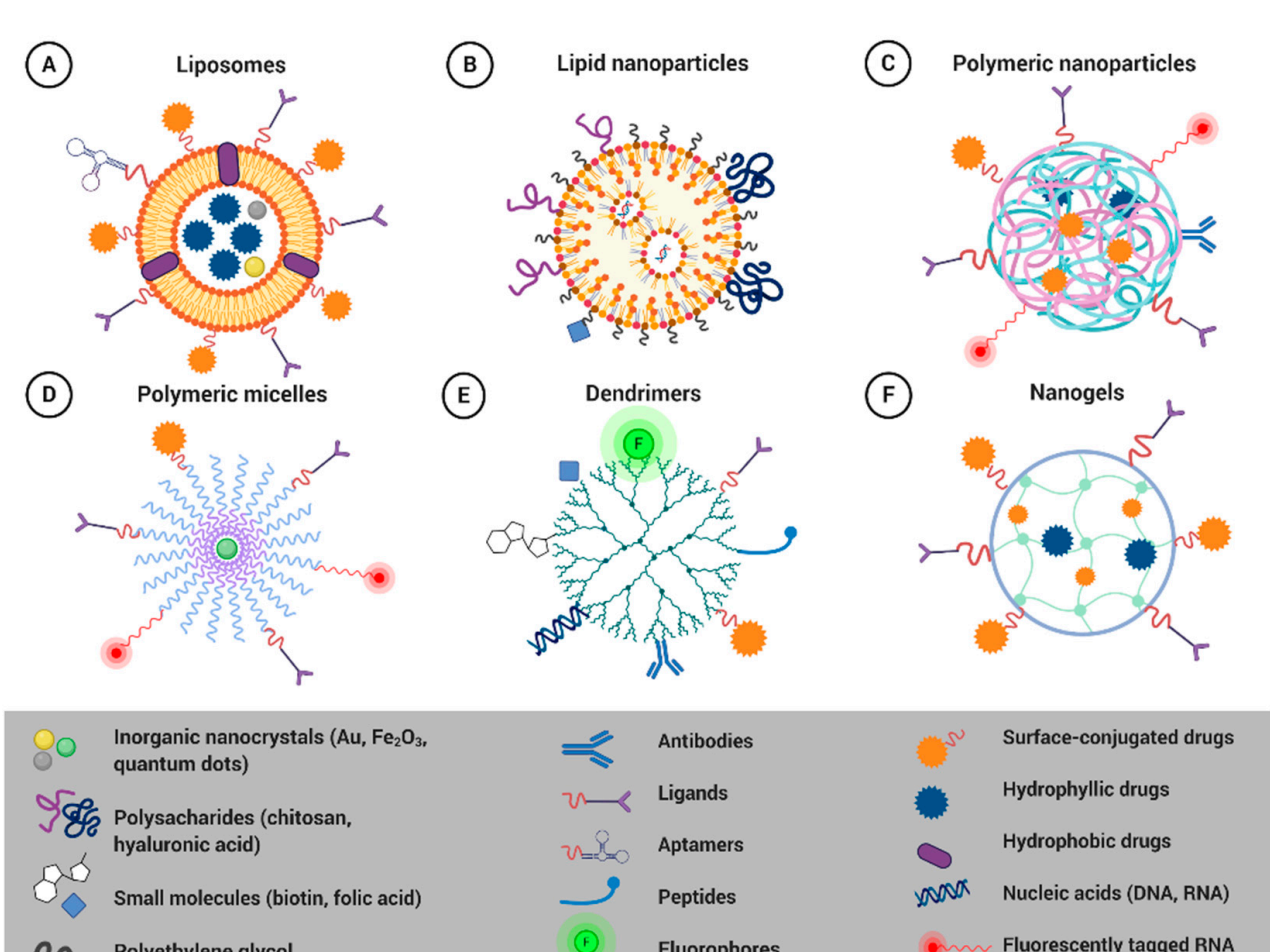
Main types of soft nanocarriers for drug delivery. Schematic examples of surface modifications and functionalization.

Nowadays, the range of nano-based delivery systems is growing [170]. Liposomes, solid lipid NPs (SLNs), polymer NPs, polymer micelles, dendrimers, nanoemulsions, and polymer-lipid hybrid NPs have been studied as promising nanocarriers for targeted drug delivery (Figure 2) [168].

With the large body of evidence accumulated over the past decades, three important elements in the development of controlled drug delivery can be defined: (1) utilization of the EPR effect for passive targeting, (2) surface modification of NPs/nanocarriers to prolong their circulation time, (3) their effective design [171]. Coating the surface of NPs with PEG (PEGylation), an FDA approved polymer, is a commonly used approach for improving the efficiency of drug and gene delivery to target cells and tissues. PEGylation protects the surface from aggregation, opsonization, and phagocytosis and prolongs systemic circulation time [172]. PEG is a hydrophilic, biocompatible, and biologically inert polymer, which is used to enhance the stability of NPs [173]. Moreover, the coating allows loading of specific ligands, antibodies, peptides, drugs, folic acid, aptamers, tumor markers, transferrin, vitamins, etc., on the surface of particles offering an exciting tool to make NPs target-specific and increase their therapeutic benefit [174,175,176]. These ligands can be easily conjugated onto PEG´s distal end via various chemical coupling strategies [177]. Cancer cells overexpress various kinds of receptors and antigens that may be used as potential drug targets in cancer therapy [178]. Entrapment of the fluorescent compound in the NPs platform allows tracking the fate of NPs in vitro and monitoring of the accumulation of the drug in the tumor and the treatment efficacy [179]. The Quantum Dots (QDs) are fluorescent semiconductor nanocrystals of about 10 nm, composed of a core coated with an envelope. QD-containing NPs have found applications in cancer medical imaging, cancer cell tracking, cancer photodynamic therapy, and cancer diagnosis [180].

#### 4.1.1. Liposomes

Liposomes (Figure 2A) are the most utilized nanomaterial for drug delivery, with many clinical products currently available [181]. Liposomal vesicles consist of amphiphilic poly-molecular lipid compounds that assemble into bi-layered self-closed spherical NPs [182]. Different structural modifications of polar and non-polar parts of phospholipids have allowed the development of various synthetic lipids with improved properties [183]. The advantage of liposomes is their ability to deliver both hydrophilic and hydrophobic drugs. Surface functionalization of liposomes enhances their stability and facilitates targeted drug, gene, or imaging agent delivery, even across the biological and physiological barriers [172,175,176,184,185,186]. They are biocompatible, of low immunogenicity, and they increase the solubility of a wide range of chemotherapeutics [187]. Several liposomal formulations, namely Doxil^®^/Caelyx™ (pegylated liposomal doxorubicin), Myocet^®^ (non-pegylated liposomal doxorubicin), DaunoXome^®^ (liposomal daunorubicin), Marqibo^®^ (liposomal vincristine), Mepact^®^ (liposomal mifamurtide), and Onivyde^®^ (pegylated liposomal irinotecan) have already been EMA and/or FDA-approved for the treatment of various cancer types, including BC [164,188].

To improve the efficacy of HDACI in BCs and other solid tumors, PEGylated liposomes were prepared for encapsulation of trichostatin A (TSA), CG1521 (CG), and PXD101 (PXD) (Table 2) [189]. These liposome formulations are promising nanocarriers as they remained stable in size, charge, and biological activity for one month when stored at 4 °C. Wang et al. utilized the iron complexation technique on VOR and LAQ824 before encapsulating them in PEGylated liposomes [190]. This strategy resulted in an improved aqueous solubility of these drugs as well as liposomal encapsulation efficiency.

#### 4.1.2. Solid Lipid Nanoparticles

SLNs (Figure 2B) consist of a hydrophobic lipid core composed of lipids (e.g., fatty acids, steroids, waxes, or triglycerides) that are in the solid state at room temperature. The lipid core is stabilized by surfactants (emulsifiers) to prevent particle agglomeration [206]. The advantage of SLNs is the easy entrapment of hydrophobic drugs in the lipidic matrix, while the outer hydrophilic shell allows the conjugation of hydrophilic drugs to lipidic components. The solid state of the lipid permits better-controlled drug release and improves the stability of the drug. Moreover, SLNs can be administered by various routes, including systemic injection, oral, transdermal, pulmonary, and ocular application [168]. SLNs can be modified to exhibit various advantages over liposomes and polymeric NPs; they are non-toxic, biocompatible, biodegradable, and highly stable [207].

Lipid-based nanostructures as carriers of the potential oral delivery of DAC were designed and synthesized by Neupane et al. [191] An ex vivo gut permeation study showed a nearly four-fold increment in the drug´s permeation from the nanostructured lipid carrier compared with the plain DAC solution and better accumulation of DAC encapsulated in lipid 4 h after oral administration compared with i.v. injection of free DAC into the tumor. Based on these results, the authors suppose that lipid-based nanocarrier systems represent a promising cancer cell treatment strategy through oral delivery. Solid lipids also represent promising nanocarriers for HDACI delivery. Encapsulation of VOR into solid lipid NPs enhanced its bioavailability and reduced the drug clearance rate compared to free VOR in vivo, whether administered orally or i.v. and increased VOR’s toxicity in multidrug-resistant BC cells in vitro [193,194,208,209]. Cyclin-dependent kinase 4 (CDK4) siRNA packaged in a lipid NP (LNP)-based delivery system that consists of an ionizable cationic lipid, helper lipid, and polyethylene glycol (PEG)-lipid showed a 16-fold increase in intracellular uptake of siRNA by BC cells and a significant G1 cell cycle arrest due to efficient down-regulation of CDK4 at both mRNA and protein levels [210]. The ncRNA nano-based drug patirisan (Onpattro^®^) has recently been approved by the FDA (in 2018) to treat polyneuropathy. It is the first clinically approved example of an RNAi therapy-delivering NP administered intravenously (i.v.) and the first FDA-approved RNAi therapeutic in general [211]. ALN-18328, the active ingredient of patirisan, is formulated as a lipid nanoparticle to targeted delivery to hepatocytes.

#### 4.1.3. Polymeric Nanoparticles

Polymeric NPs (Figure 2C) are the simplest form of soft-materials for nanobiomedical applications [212]. They can be composed of synthetic biodegradable polymers, some of which were already approved by the FDA for tissue engineering scaffolds and drug delivery vehicles [213]. Alternatively, they can be made of natural polymers such as chitosan, gelatin alginate, and albumin. The advantages of polymeric NPs include the capacity to encapsulate both hydrophilic and hydrophobic drugs and straightforward activation with specific molecules for targeted delivery to the tumor mass [214]. While synthetic polymers allow sustained drug release, within a period of days to weeks, natural polymers are more easily and rapidly degraded [215]. Polymeric NPs are stable, bioavailable, have prolonged circulation time in peripheral blood, enable controlled drug release, and, compared to liposomes, they possess superior drug loading capacities [216]. There are several FDA-approved polymeric nano-drugs, such as Adagen^®^, Cimzia^®^, Macugen^®^, Neulasta^®^, Pegasys^®^, PegIntron^®^, Renagel^®^, and Somavert^®^ [212].

An innovative aspect of the nano-based delivery platform is the conjugation of CI-994 (tacedinaline) through a clickable acid-responsive linker to a macromonomer. Such a prodrug monomer could be polymerized to create a formulation that releases the HDACI in a pH-dependent manner while minimizing in vivo drug release during blood circulation [195]. A pendant SAHA derivatized polymer able to form spherical micelles is a promising novel prodrug carrier. This nanoformulation retains the pharmacological activity of SAHA, inhibits the proliferation of tumor cells, and induces histone acetylation [196]. Belinostat-loaded polymer NPs, decorated with a novel cell-penetrating polymer, poly(guanidinium oxanorbornene), significantly enhanced intracellular uptake, decreased the tumor volume, and increased the intratumoral acetylation of histone H4 [197]. The safety of orally administered SAHA-loaded polymer NPs manifested low cytotoxicity in lung cancer cells and favorable biodistribution patterns in vivo [198]. A VOR nanofiber-coated stent was shown to be a promising candidate for cholangiocarcinoma treatment [217]. Using the emulsion-solvent diffusion technique, Alp et al. have developed a biocompatible starch NP formulation for delivery of CG-1521 to hard-to-treat BCs [218]. This formulation improves the bioavailability and half-life of this HDACI without affecting the drug´s mechanism of action.

A stable nanosized ribophorin II (RPN2) siRNA–atelocollagen complex was prepared to improve the in vitro and in vivo resistance of BC cells to DOX and taxanes. RPN2 silencing caused reduced glycosylation of the P-glycoprotein in vitro and markedly reduced tumor growth in vivo [219].

#### 4.1.4. Polymeric Micelles

Polymeric micelles (Figure 2D) are supramolecular delivery systems composed of multiple amphiphilic block copolymers, self-assembling in aqueous environments at a defined concentration [206]. The micelles´ core-shell structure presents a hydrophobic core and the outer hydrophilic shell. The core can be tailored for the controlled delivery of hydrophobic drugs, while hydrophilic drugs can be adsorbed or chemically linked to the shell [9]. Polymeric micelles have high stability and exhibit prolonged circulation time in the blood [168]. Poly(ethylene glycol)–poly(lactic-co-glycolide) (PEG–PLGA) di-block copolymer micelles represent one of the most promising biocompatible platforms for drug delivery [220]. Lipids can also be used to form the hydrophobic core of these hybrids for encapsulation of many types of poorly water-soluble drugs [221]. FDA approved polymeric micelles, already used in clinical practice, include Copaxone^®^, Eligard^®^, Estrasorb™, Oncaspar^®^, Krystexxa^®^, Plegridy^®^, and Adynovate^®^ used for the treatment of sclerosis, prostate cancer, and blood malignities [212].

The micelles are predominantly utilized as versatile nanocarriers for the delivery of DNMT inhibitors. Encapsulation of AZA or DAC into micelles has increased their stability under physiological conditions, markedly enhanced their therapeutic efficacy, provided controlled pH-dependent drug release, significantly down-regulated DNMT1 and DNMT3b expression, and increased expression of caspase-9 in murine xenograft models of BC [199,200]. Intelligent DAC-loaded micelles have been prepared by inserting cancer-specific gelatinase-cleavable peptide between two polymers. These micelles manifested superior cellular uptake in the tumor-bearing xenografts due to an active (gelatinase-stimuli) targeting strategy and more efficient gene demethylation compared with their counterparts lacking gelatinase features [201,202]. DAC-loaded hybrid lipid-polymer micelles were efficiently internalized by BC cells and rescued the expression of silenced tumor suppressor genes in cancer cells [192]. Several micelle formulations have been proposed and tested as a promising nanocarrier for HDAI delivery. Kwak et al. revealed higher efficacy of tumor growth inhibition, enhanced drug accumulation, and higher inhibition rate of HDAC expression in a xenograft mice model by VOR-encapsulated micelles compared with the free drug [203]. Similarly, higher therapeutic efficacy was confirmed by VOR- or quisinostat-loaded hybrid lipid-polymer micelles compared with free HDACIs [204]. These micelles suppressed DNA double-strand break repair in tumor cells and showed a synergistic effect in mouse xenograft in vivo models. Micelles loaded with antisense-miR-21 and antisense-miR-10b were prepared to block the functions of endogenous miRNAs, regulating genes involved in cell proliferation, differentiation, and apoptosis. Treatment of mice bearing subcutaneous tumor xenografts with this formulation resulted in a substantial tumor growth reduction, thus indicating a potential new therapeutic approach for triple-negative BC [222].

#### 4.1.5. Dendrimers

Dendrimers (Figure 2E) are three-dimensional, hyper-branched, or tree-like polymers having a central core with repeated branches of interior layers and exterior terminal functionality. These structures offer high availability and a wide range of molecular moieties for the chemical conjugations and internal cavities to encapsulate drugs and nucleic acids [223]. Dendrimers can be used to deliver various molecules (therapeutics, imaging, and targeting agents) in a single particle due to the presence of different functional monomers [220]. Dendrimers are suitable drug-delivery systems because of their nanometric size, ease of fabrication, monodispersity, lipophilicity, multidrug loading capacity, controlled drug release, low toxicity, biocompatibility, and ability to easily penetrate cell membranes [9]. Among different commercially available dendrimers, diaminobutyric polypropylenimine (DAB), polyamidoamine (PAMAM), poly (amidoamine-organosilicon) (PAMAMOS), poly (Lysine), and poly (propylene imine) (PPI) are the most promising platforms for delivering cargos to target sites [223].

Finlay et al. used a modified poly (amidoamine) (PAMAM) dendrimer for delivery of siRNA, targeting the TWIST1 transcription factor [224]. *TWIST1* is often overexpressed in aggressive BCs and is involved in regulating cell migration through EMT. These PAMAM-siRNA complexes significantly down-regulated *TWIST1* and EMT-related target genes in vitro as well as in xenograft orthotopic tumors. PAMAM dendrimer was also used to deliver antisense oligonucleotides targeting the vascular endothelial growth factor VEGF-ASODN to inhibit the tumor vascularization of breast tumor tissue using a human breast tumor xenograft mice model [225].

#### 4.1.6. Nanogels

Nanogels (Figure 2F) are three-dimensional polymeric networks with a high capacity for water uptake. They are mostly made of synthetic polymers or biopolymers, which contain hydrophilic and hydrophobic monomers, cross-linked by either physical or chemical bonds, which influence many network properties, like swelling, elastic modulus, and transport properties [226,227]. In aqueous media, nanogels form semi-solid states (hydrogels). The porous network allows high-drug entrapment efficiency. Nanogels can carry various drugs with low or high molecular weight and either hydrophilic or hydrophobic. The advantages of nanogels include high drug encapsulation capacity, enhanced drug stability, minimal toxicity, biocompatibility, prolonged blood circulation time, and stimulus responsiveness to drug release (pH, magnetic field, light, ionic content, and temperature) [206].

Nanogels (NGs) have also been explored for treating solid tumors. DAC-loaded nanogels decorated with PEG manifested sustained DNMT1 depletion, prolonged cancer cell arrest in the G2/M cell-cycle phase, and significantly enhanced the antiproliferative effect of DAC [205].

### 4.2. Nanoplatforms for Combination Therapy

A promising strategy to achieve more significant therapeutic benefits appears to be the combination therapy involving epi-drug-loaded NPs and either a chemotherapeutic-encapsulated nanocarrier or free standard anticancer drugs, or co-packaging of epi-drugs and anticancer drugs in one nanoplatform.

Li et al. have recently shown that combination therapy with low-dose DAC-loaded NPs and DOX-loaded NPs is more effective and opens up new possibilities for the management of BC [200]. In line with these results, Vijayaraghavalu et al. have revealed that a combination of epigenetic drugs (DAC + SAHA) encapsulated in biodegradable NGs more effectively overcomes drug resistance than the same drugs in solution [228]. Moreover, pretreatment with epigenetic drugs in nanogels, then with DOX in nanogels, was most effective in overcoming resistance even at low doses of DOX. Co-packaging DAC and arsenic trioxide (ATO) into alendronate-conjugated bone-targeting hybrid lipid-polymer micelles should enhance the synergistic effect of DAC and ATO in the treatment of MDS and reduce systemic toxicity of chemotherapeutics [229]. Using co-axial and multi-needle electrohydrodynamic atomization (EHDA) technology, Parhizkaz et al. have co-encapsulated DAC and cisplatin in a single PLGA-based nanocarrier [230]. The EHDA technique enables fine-tuning of individual drug release characteristics, with rapid DAC and slower cisplatin release, thus achieving the maximum synergistic therapeutic effect and overcoming the chemoresistance to cisplatin. Lysophosphatidic acid receptor 1 (LPAR1)-targeted lipid nanoemulsions (nanoscale oil-in-water emulsions) were developed for encapsulation of two drugs with different chemistries, DAC (hydrophilic) and panobinostat (PAN–HDACI, hydrophobic) and targeted co-delivery in triple-negative BC tissues [231].

Pendant SAHA derivatized polymer (POEG-b-PSAHA) micelles were used as nanocarriers for DOX delivery. A DOX/POEG-b-PSAHA formulation resulted in an improved therapeutic effect in vivo compared to free DOX, Doxil, or POEG-b-POM-loaded DOX micelles, indicating that SAHA-based prodrug micelles may serve as a dual functional carrier for combination strategies in epigenetic-oriented anticancer therapy [196]. Ruttala et al. developed a transferrin-anchored albumin nanoplatform with PEGylated lipid bilayers (Tf-L-APVN) for the targeted co-delivery of paclitaxel and VOR in solid tumors [232]. At in vitro conditions, Tf-L-APVN significantly enhanced the synergistic effects of paclitaxel and VOR on the proliferation of breast and liver cancer cells. In HepG2 tumor-bearing mice, the co-delivery of paclitaxel and VOR in one nano-based platform significantly inhibited the tumor growth, thus offering great potential in the chemotherapy of solid tumors.

Using a layer-by-layer approach, Deng et al. generated a siRNA-loaded film superimposed on a simple DOX-loaded liposome [233]. They showed that combination therapy with siRNA targeting multidrug resistance protein 1 (MRP1) significantly enhanced DOX efficacy in vitro, and in vivo resulted in a substantial reduction in the tumor of tumors, which otherwise are nonresponsive to treatment with DOX.

## 5. Biosafety of Soft Nanocarriers

Despite the many advantages of using nano-based delivery systems, there are also significant drawbacks that must be considered, as they might present a real challenge for clinical use [186]. Currently, there is very little regulatory guidance in the field of biomedical applications of NPs [234]. Although the EPR effect has been described as a basis for successful passive targeting of tumors, the complexity of this process must also be highlighted. The EPR effect can be influenced by the interactions of NPs with the biological systems, including proteins, blood flow, and the tumor microenvironment. Nanoparticle properties, including size, shape, surface properties, porosity, and structure, can affect the EPR effect [235]. For example, it has been reported that the high interstitial fluid pressure in tumors can hinder the accumulation of nanocarriers since the high density of the extracellular matrix can reduce the chances of NP penetration [236].

It is also difficult to predict the behavior and responses of nanocarrier interactions with biological systems during the drug delivery process [220]. For instance, the interaction of NPs with the renal system and its role in blood clearance is a crucial aspect to be taken into account. An efficient bloodstream clearance is important to avoid NPs accumulating, which might lead to adverse long-term effects. The efficiency of kidney clearance has been reported to be affected by small changes in the size of the NPs. At the same time, unspecific clearance, possibly leading to drug release far from the tumor site, must also be avoided [237,238].

Thus, the nanocarriers´ possible toxicity must be taken into account and thoroughly investigated [239]. Nanomaterials pose particular challenges in the evaluation of their toxicity in comparison with chemical substances. Besides the chemical composition, the particles physical properties (e.g., their size, shape, agglomeration/aggregation, solubility/dispersibility, surface charge, redox potential) play an important role in eliciting biological responses [240]. In this sense, a proper hazard and risk assessment needs to be applied. This means that external and internal exposures, as well as cellular uptake of the NPs, must be defined. In order to investigate all the possible pathways through which toxicity might arise, a battery of relevant toxicity assays should be applied. As NPs have been reported to de-regulate gene expression and affect epigenetic mechanisms involved in several biological processes, including cancer development [44,165], these effects have to be taken into consideration in addition to cytotoxicity and genotoxicity.

Attention must also be given to the choice of exposure models. Although the use of in vivo models cannot yet be completely excluded, alternative in vitro tests can be valid substitutes for initial screenings of cyto- and genotoxicity and for investigating the underlying molecular mechanisms of toxicity of nanomaterials. More advanced and relevant in vitro models to assess efficacy and safety have been developed, moving beyond the traditional 2D monocultural models, towards 3D, e.g., spherical tumor models, and more complex multicellular models, which better resemble and mimic real-life conditions [241]. Several nanomaterials have not yet had their potential adverse biological effects fully assessed due to costs and time constraints associated with the experimental assessment, frequently involving animals [242].

There are OECD test guidelines for in vitro assays adapted to the testing of NPs. In vitro assays are usually robust, fast, and cost-effective compared to in vivo methods, and several high-throughput screening methods for relevant cells from humans and other mammals are available to study the effects of NPs [243,244]. Nowadays, in vitro assays adapted for testing nanomaterials are performed in a controlled manner, taking physicochemical characterization and cellular uptake into consideration. However, such tests need further validation because of putative NM-induced interferences [245].

To fully understand the advantages and disadvantages of NP-based therapeutics, more clinical data are needed to identify the best applications for nano-chemotherapeutics [13]. In the field of cancer nanomedicine research, it is important to take into account the complex heterogeneity within and among human tumors and to focus on the design of precision nanomedicines to achieve personalized cancer treatments [246]. In this sense, understanding which physicochemical properties are coupled with adverse effects is critical for designing safer and more effective nanomaterials for cancer treatment. This so-called “Safe(r)-by-Design” approach requires the implementation of safety evaluation early in the development of nanomaterials [247], together with understanding their toxicokinetics and modulation of the immune system [248,249,250].

## 6. Conclusions

In BC, there are tumor subtypes, which are considered to be hard to cure, and do not respond to standard therapeutic agents, and have a high recurrence probability. In recent decades, the intense study of epigenetic deregulation in many cancers has uncovered the potential of new therapeutic approaches, targeting reversible changes in the epigenome. The limited tolerability, low efficacy, and off-target effects of most epi-drugs remain significant challenges in this experimental approach as applied to solid tumors. The exploration of lower doses, sequential scheduling, and their targeted delivery might considerably improve the therapeutic index. In this regard, nanotechnology has revolutionized the field of drug delivery for epigenetic therapy of solid tumors. Different formulations of soft nanocarriers have been employed to increase the stability, solubility, and specificity of DNMT and histone inhibitors as well as non-coding RNAs. In addition, conventional chemotherapeutic agents have been successfully co-packed with epi-drugs in nanocarriers for combination therapy. Data from preclinical and clinical trials of nano-based epi-drugs for BC treatment demonstrate reduced systemic toxicity and improved efficacy compared to conventional free-drug formulations.

Furthermore, nanomedicine offers the possibility to refine the detection, diagnosis, and conventional therapy of many cancers by combining different agents in a single multi-component nano-drug. Although promising, nano-based delivery systems should follow the Safe(r)-by-Design approach and be rigorously tested at all phases of development to prevent adverse health effects and environmental hazards.

The use of nano-based delivery systems, together with the benefits of a new generation of epi-drugs and the emergence of robust biomarker data, will help to advance personalized-targeted therapy and the efficacy of treatment for breast and other cancers.

## Figures and Tables

**Table 1:** Epigenetic drugs in clinical trials focused on breast cancer.

Epi-Drug	Other Interventions	Status	Phase	Trial No.
Decitabine	LBH589, Tamoxifen	Terminated	1, 2	NCT01194908
	Paclitaxel	Unknown	1b	NCT03282825
	Carboplatin	Recruiting	2	NCT03295552
	Doxorubicin and 4 more	Recruiting	2	NCT02957968
Azacitidine	Nab-paclitaxel	Completed	1, 2	NCT00748553
	Entinostat	Active, not recruiting	2	NCT01349959
	Definitive breast	Withdrawn	NA	NCT01292083
	Fulvestrant	Terminated	2	NCT02374099
	Durvalumab	Active, not recruiting	2	NCT02811497
Valproic acid	FEC100	Terminated	2	NCT01010854
	Hydralazine	Terminated	2	NCT00395655
	Bevacizumab, Cetuximab	Recruiting	1	NCT01552434
Entinostat	Capecitabine	Recruiting	1	NCT03473639
	Exemestane	Recruiting Completed Active, not recruiting Active, not recruiting	3 2 3 2	NCT03538171, NCT00676663, NCT02115282, NCT03291886
	Fulvestrant	Withdrawn	2	NCT02115594
	Lapatinib Ditosylate, Trastuzumab	Completed	1	NCT01434303
	Anastrozole	Terminated	2	NCT01234532
	Nivolumab, Ipilimumab	Active, not recruiting	1	NCT02453620
	Atezolizumab and 6 more	Recruiting	1, 2	NCT03280563
Romidepsin	Cisplatin, Nivolumab	Suspended	1, 2	NCT02393794
	Abraxane	Terminated	1, 2	NCT01938833
	Alone	Completed	2	NCT00098397
Vorinostat	Olaparib	Not yet recruiting	1	NCT03742245
	Paclitaxel and 3 more	Completed	1, 2	NCT00574587
	Alone	Completed Terminated Completed Withdrawn Completed Terminated	1 2 2 NA 1 2	NCT00719875, NCT00132002, NCT00262834, NCT01695057, NCT00788112, NCT00126451
	Tamoxifen	Completed Terminated	2 2	NCT00365599, NCT01194427
	Tamoxifen, Pembrolizumab	Terminated Not yet recruiting	2 2	NCT02395627, NCT04190056
	Carboplatin, Nab-paclitaxel	Active, not recruiting	2	NCT00616967
	Ixabepilone	Completed	1	NCT01084057
	Lapatinib	Terminated	1, 2	NCT01118975
	Paclitaxel, Bevacizumab	Completed	1, 2	NCT00368875
	Anastrozole, Letrozole, Exemestane	Completed Completed	NA NA	NCT01720602, NCT01153672
	Trastuzumab	Completed	1, 2	NCT00258349
Belinostat	Ribociclib	Not yet recruiting	1	NCT04315233
Panobinostat	Alone	Completed Terminated Withdrawn	2 2 1	NCT00777049, NCT00777335, NCT00993642
	Trastuzumab	Terminated	1, 2	NCT00567879
	Letrozole	Completed	1, 2	NCT01105312
	Trastuzumab, Paclitaxel	Completed	1	NCT00788931
	Capecitabine, Lapatinib	Completed	1	NCT00632489

**Table 2:** Nanoplatforms for epigenetic drug delivery.

Nanocarrier	Loaded Drug	Reference
PEGylated liposomes	trichostatin A, CG1521, and PXD101	[189]
PEGylated liposomes with Fe complex	VOR and LAQ824	[190]
Hybrid lipid-polymer NPs	DAC	[191,192]
Solid lipid NPs	VOR	[193]
Solid lipid NPs decorated with hyaluronic acid	VOR	[194]
Norbornene polyethylene oxide macromonomer	CI-994 (tacedinaline)	[195]
POEG blocks	VOR	[196]
PLGA NPs decorated with PGON	belinostat and VOR	[197,198]
PLGE-PEG nano-micelles	AZA	[199]
PEG-PLA di-block copolymer	DAC	[200]
Gelatinases-stimuli di-block copolymers (PEG, PCL)	DAC	[201,202]
LGE block copolymer	VOR	[203]
Lipid-polymer (DSPE-PEG-COOH-PLGA-lecithin-PEG) core-shell NPs	VOR and quisinostat	[204]
Nanogels	DAC	[205]
